# Compression Therapy in the Prevention of Postthrombotic Syndrome: A Systematic Review and Meta-Analysis

**DOI:** 10.1097/MD.0000000000001318

**Published:** 2015-08-07

**Authors:** Hong-Tao Tie, Ming-Zhu Luo, Ming-Jing Luo, Ke Li, Qiang Li, Qing-Chen Wu

**Affiliations:** From the Department of Cardiothoracic Surgery (H-TT, QL, Q-CW), The First Affiliated Hospital of Chongqing Medical University; Division of Immunology (M-ZL, M-JL), The Children's Hospital of Chongqing Medical University; and Department of Orthopedics Surgery (KL), The First Affiliated Hospital of Chongqing Medical University, Chongqing, China.

## Abstract

Although compression therapy has been widely used after deep vein thrombosis (DVT), its efficacy in prevention of postthrombotic syndrome (PTS) remains disputable. We aimed to update the meta-analysis to comprehensively evaluate the effect of compression therapy on the prevention of PTS in adult patients after DVT.

PubMed, Embase, and Cochrane library databases were systematically searched. Randomized controlled trials (RCTs) evaluating the preventive effect of compression therapy on PTS in adult patients after DVT were included. The primary outcome was the incidence of PTS. All meta-analyses were performed using random-effects models regardless of the heterogeneity. Subgroup and sensitivity analysis were also performed to examine the robustness of the pooled effects according to our predesigned plan. Potential publication bias was assessed.

Eight RCTs with 1598 patients were included. Overall, compression therapy could significantly reduce the incidence of PTS (estimate 0.68, 95% confidence interval [CI] 0.52–0.90; *P* = 0.007). However, it was only associated with a reduction in the incidence of mild/moderate PTS (relative risk [RR] 0.66, 95% CI 0.46–0.93; *P* = 0.019) but not in the incidence of severe PTS (RR 0.64, 95% CI 0.27–1.50; *P* = 0.31). Additionally, compression therapy failed to reduce the incidence of recurrent venous thromboembolism (RR 0.91, 95% CI 0.65–1.27; *P* = 0.58), the incidence of ulceration (RR 0.74, 95% CI 0.36–1.53; *P* = 0.42), or mortality (RR 0.99, 95% CI 0.72–1.37; *P* = 0.96). No publication bias was observed.

Current evidence still supports compression therapy to be a clinical practice for prophylaxis of PTS in adult patients after DVT. However, our findings should be cautiously interpreted because of heterogeneity and hence more large-scale and well-designed RCTs are still warranted.

## Abstract 2

Supplemental Digital Content is available in the text

## INTRODUCTION

Postthrombotic syndrome (PTS) is increasingly recognized to be a frequent long-term complication of deep vein thrombosis (DVT). It is characterized by chronic, persistent pain, swelling, and even venous ulcers in severe cases,^1,2^ and the diagnosis of PTS is generally deferred after 6 months.^1^ The prevalence of PTS is reported to vary from 20% to 81.8%^3–10^ and 5% to 23.5% of them are severe cases.^3,6,10^ The variation of the incidence is partly due to different follow-ups and it is reported that the prevalence increases as time goes on.^1^ PTS is associated with poorer health-related quality of life, limited daily social and physical activity, and psychological distress.^11,12^ Moreover, the average health care cost of DVT accompanied with PTS was estimated to be >10 times higher than DVT only.^13^ Furthermore, it was estimated that 2 million workdays were lost annually in the United States resulting from PTS-induced leg ulcers.^14,15^ Given its considerable prevalence and socioeconomic burden, the prevention of PTS is of great importance.

Compression therapy, a noninvasive method of wearing elastic compression stockings or bandages on the affected leg, was reported to improve microcirculation and prevent PTS by reducing venous hypertension and reflux in patients with DVT.^16,17^ Consistently, the beneficial effect of compression therapy was confirmed by several clinical trials^3,5,6^ and meta-analyses.^18,19^ However, this seductive finding was not validated by subsequent studies.^4,7–10^ With increasing published evidences,^9,10^ we decided to update the meta-analysis of randomized controlled trials (RCTs) to solve the disputes and comprehensively evaluate the effect of compression therapy on prevention of PTS in patients with DVT.

## METHODS

This systematic review and meta-analysis was performed and reported according to Preferred Reporting Items for Systematic Reviews and Meta-Analyses (PRISMA) (Additional file 1, http://links.lww.com/MD/A356).^20^ As all analyses were performed based on previous published researches, the ethical approval and patient consent are not required. Two investigators independently conducted the literature search, data extraction, and quality assessment. Any disagreements were solved by discussion.

### Search Strategy and Inclusion Criteria

PubMed, Embase, and Cochrane Library were searched (updated from the inception to June 2015) by combining text words and subject terms. No additional limitation was imposed, and the detailed search strategy was shown in an additional DOC file (Additional file 2, http://links.lww.com/MD/A356). The references of identified articles and relevant reviews were manually checked via full-text screening.

Inclusion criteria included the following: Study populations: adult patients undergoing DVT; Intervention: compression therapy; Control: placebo or no treatment; Outcome: the incidence of PTS; and Study design: RCT.

### Data Extraction and Outcome Measures

The following information was extracted by using the predesigned forms: first author, year of publication, sample size, characteristics of patients, cointervention, intervention of compression therapy, control, interval between diagnosis and treatment, follow-up, diagnosis criteria of PTS, the incidence of PTS, the incidence of mild/moderate PTS, the incidence of severe PTS, the incidence of recurrent venous thromboembolism, the incidence of ulceration, and the mortality. Corresponding authors were contacted, in case essential data were unavailable. In addition, the hazard ratio (HR) was obtained from the survival curve with the method supplied by Tierney et al^21^ if necessary.

The primary outcome was the incidence of PTS, including mild/moderate PTS and severe PTS. Secondary outcomes included the incidence of recurrent venous thromboembolism, the incidence of ulceration, and the mortality.

### Risk of Bias Assessment

The quality of included studies was assessed in accordance with Cochrane Collaboration's tool for risk of bias assessment,^22^ which covers 6 aspects as follows: selection bias (random sequence generation, allocation concealment), performance bias (blinding of participants, blinding of personnel), attrition bias (incomplete outcome), detection bias (blinding of outcomes assessments), reporting bias (selective reporting), and other potential source of bias. Each item was rated as low, high, or unclear risk, and the overall risk of bias of a study was concluded by summarizing all the 6 aspects. The summary risk of bias was considered to be low (low risk in all domains), high (high risk in one or more domains), or unclear (low or unclear risks in all domains).

### Statistical Analysis

For the primary outcome, the effect of compression therapy, compared with placebo or no treatment, was expressed as relative risk (RR) with 95% confidence interval (CI) or HR with 95% CI in the included trials. For the secondary outcomes, differences were all expressed as RR with 95% CI. Suffering less from selection bias in relation to the endpoints, HR was treated as RR and preferred to be used in the meta-analysis when combining the effect sizes (ESs). Random-effects models were used in all meta-analyses regardless of the heterogeneity. The inconsistency across studies was tested by using the *I*^2^ statistic and *Q* test. *I*^2^ statistic represents the proportion of variation on account of the heterogeneity instead of chance and is perceived to be low (25% ≤ *I*^2^ < 50%), moderate (50% ≤ *I*^2^ < 75%), and high (*I*^2^ ≥ 75%). *I*^2^ ≥ 50% and *Q* test with *P* < 0.10 suggested significantly high heterogeneity.^23^ To reduce the likelihood of spurious results, subgroup analyses were prespecified. Additionally, sensitivity analyses according to several exclusion criteria and by omitting one study and pooling the others in each turn were also performed. Potential publication bias was assessed by inspecting the funnel plot and statistically detected by Egger test.^24^ A 2-sided *P* < 0.05 was considered statistically significant except where it was emphasized particularly. All statistical analyses were performed with Stata 12.0 software (StataCorp, College Station, TX) except the risk of bias was evaluated by using Review Manager Version 5.1 (The Cochrane Collaboration, Software Update, Oxford, UK).

## RESULTS

### Study Identification and Selection

**FIGURE 1:**
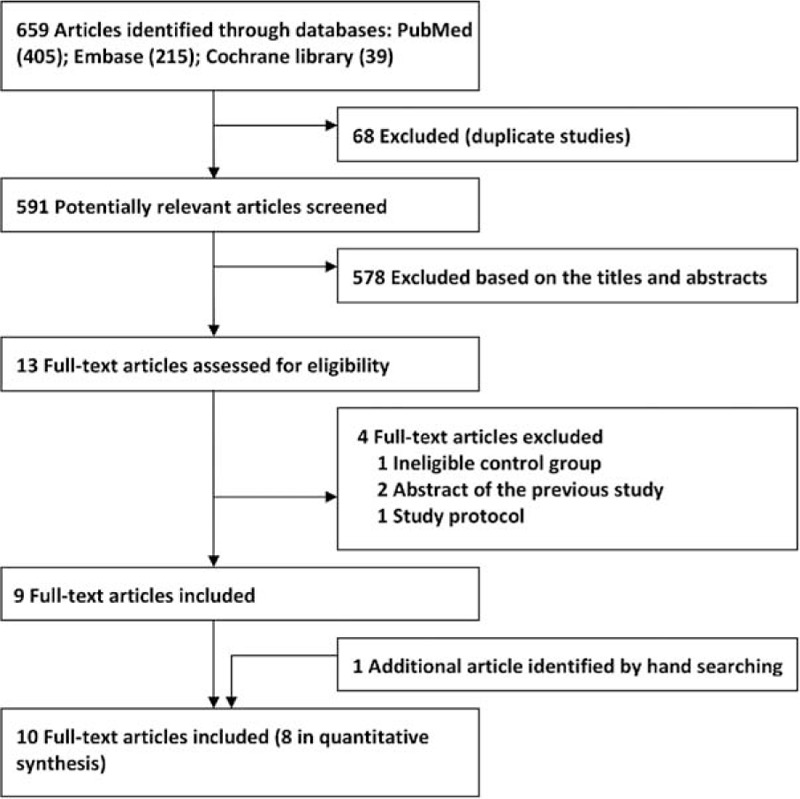
Identification process for the RCTs included in the meta-analysis. RCT = randomized controlled trial.

Six hundred and fifty-nine studies were identified by initial database search. Of them, 68 were excluded due to duplications, and 578 excluded via screening titles and abstracts. After detailed assessment of the remaining 13 studies, 4 were excluded because of ineligible control and outcome,^25^ abstract publications of the included full-texts,^26,27^ and study protocol.^28^ Additionally, one^29^ was retrieved by hand-searching. Therefore, 10 studies^3–10,29,30^ were eligible for the systematic review and 8 of them^3–10^ were included in the meta-analysis. Flow chart of the study identification process is presented in Figure 1.

### Study Characteristics

Table 1 summarizes the baseline characteristics of the 8 eligible RCTs. They were published from 1997 to 2014 and enrolled 37 to 803 patients with a total of 1598. The follow-up varied from 1 to 6.3 years and the incidence of PTS in the control was between 20% and 84.8%. Six trials^3,4,6,8–10^ only enrolled patients with a first episode of DVT, whereas the remaining two^5,7^ recruited those with a first or recurrent DVT. The comparison group was blank control in 6 studies^3,5–9^ and placebo control in the other two.^4,10^ Interval from DVT diagnosis to intervention was <48 hours,^9^ <3 weeks,^3,6,10^ 6 months,^7^ and 1 year,^4^ respectively. However, patients in 2 studies^5,8^ were immediately randomized to treatment or control group, but the intervention only lasted within the acute stage and then all patients were encouraged to receive compression therapy. The pressure of the compression therapy ranged from 20 to 40 mm Hg in all studies except one without reporting.^5^ PTS was defined in accordance to Villalta-Prandoni scale (VPS),^3,5,6,8–10^ Ginsberg criteria,^4^ or Clinical–Etiology–Anatomic–Pathophysiologic scoring system.^7^ Outcome data of each included study are supplemented in Table S1 (Additional file 3, http://links.lww.com/MD/A356).

**TABLE 1:**
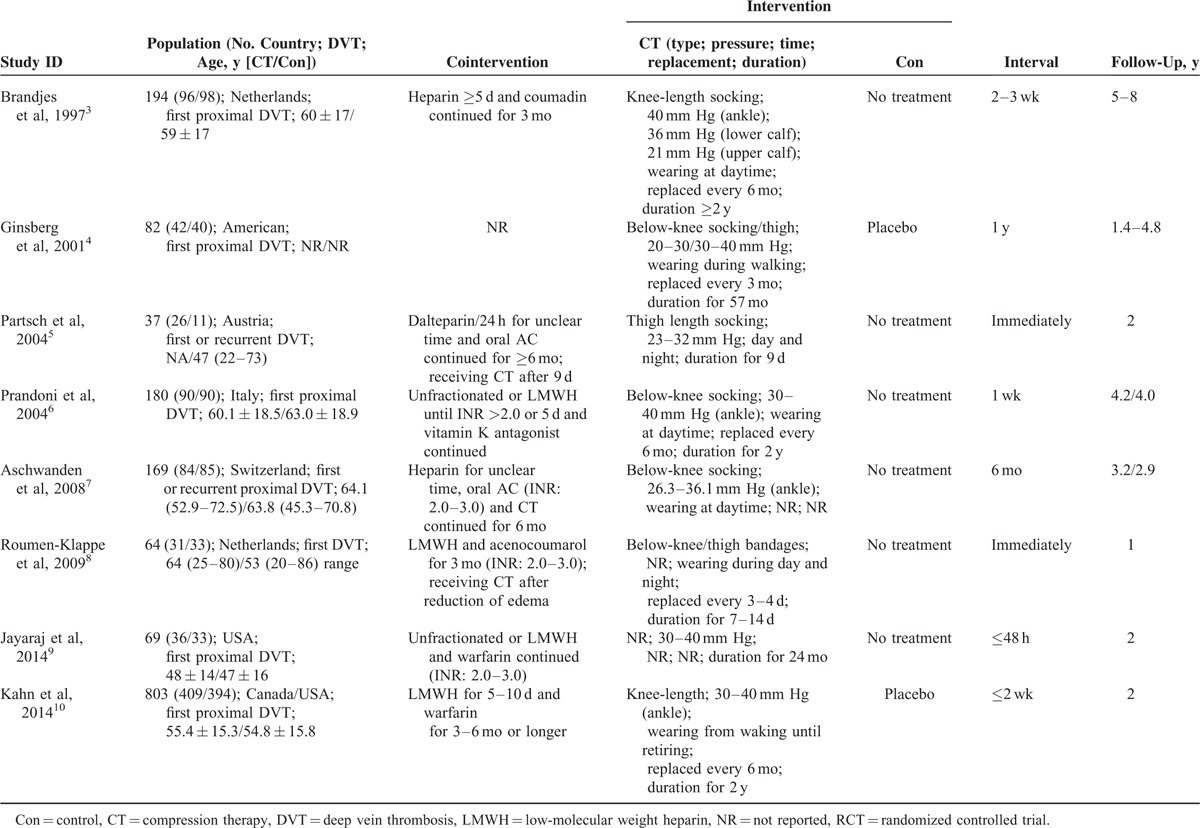
Characteristics of the 8 Included RCTs

### Risk of Bias Assessment

**FIGURE 2:**
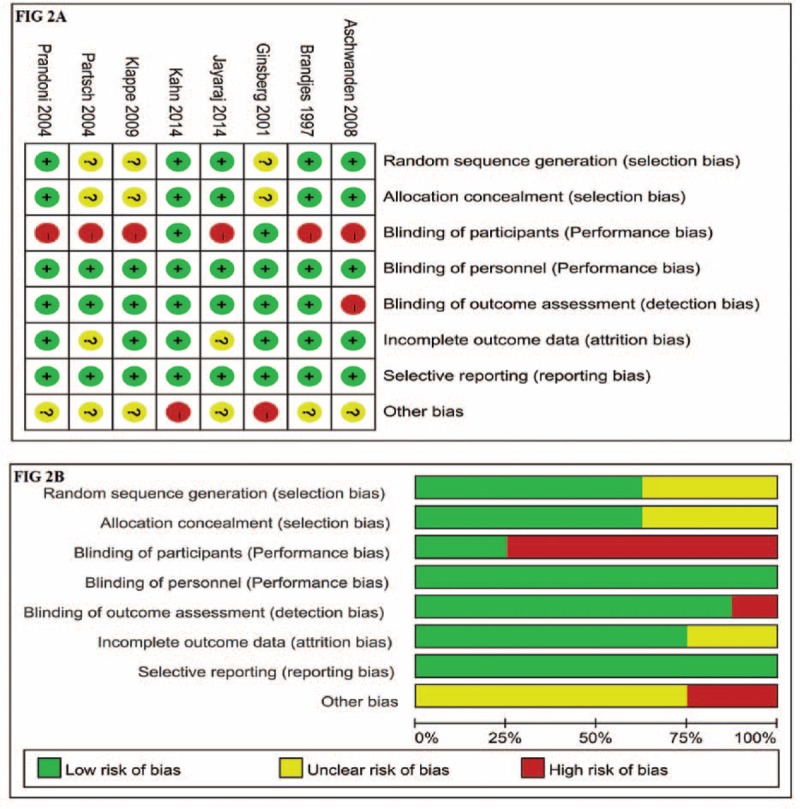
Risk of bias assessment. (A) Risk of bias summary. (B) Risk of bias graph.

Risks of bias of included studies are shown in Figure 2. Six studies^3,5–9^ had performance bias for the intervention not blinded to patients. One^7^ had detection bias because clinical follow-up examinations were done by specialists who were not blinded to treatment allocation. Additionally, other potential biases existed in 2 studies due to premature termination of recruitment^4^ and modification of the study protocol,^10^ respectively.

### The Primary Outcome: PTS

**FIGURE 3:**
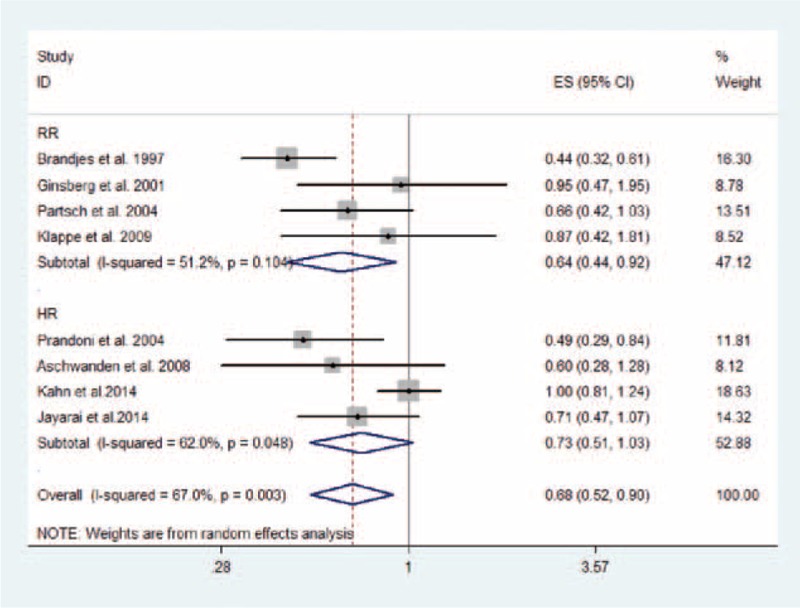
Effect of compression therapy on PTS in patients after DVT. CI = confidence interval, DVT = deep vein thrombosis, ES = estimate, HR = hazard ratio, PTS = postthrombotic syndrome, RR = relative risk.

**FIGURE 4:**
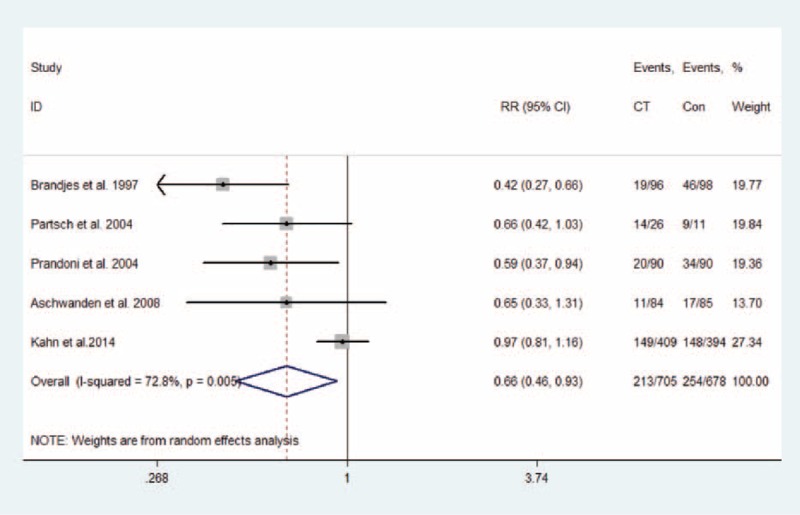
Effect of compression therapy on mild/moderate PTS in patients after DVT. CI = confidence interval, DVT = deep vein thrombosis, PTS = postthrombotic syndrome, RR = relative risk.

**FIGURE 5:**
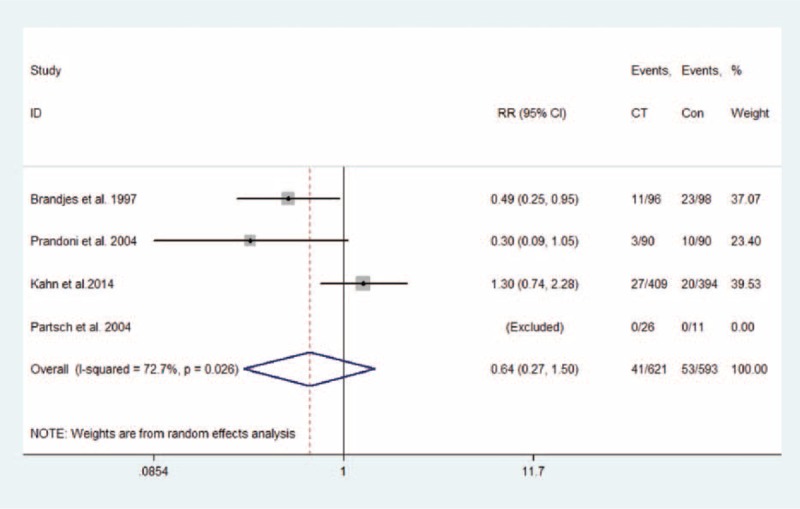
Effect of compression therapy on sever PTS in patients after DVT. CI = confidence interval, DVT = deep vein thrombosis, PTS = postthrombotic syndrome, RR = relative risk.

Eight studies, with a total of 1598 patients (814 in compression therapy group and 784 in the control group), were eligible to evaluate the effect of compression therapy on the prevention of PTS. Overall, compression therapy could significantly decrease the incidence of PTS (estimate 0.68, 95% CI 0.52–0.90; *P* = 0.007), with moderate heterogeneity (*I*^2^ = 67.0%; *P*_H_ = 0.003; Figure 3). The result remained consistent with the pooled effect by combining RRs (RR 0.73, 95% CI 0.56–0.95; *P* = 0.021; *I*^2^ = 76.5%; *P*_H_ < 0.001; Figure S1, Additional file 4, http://links.lww.com/MD/A356). However, the pooled RR of 4 studies showed that compression therapy could reduce the incidence of mild/moderate PTS (RR 0.66, 95% CI 0.46–0.93; *P* = 0.019; *I*^2^ = 72.8%; *P*_H_ = 0.005; Figure 4) but not the incidence of severe PTS (RR 0.64, 95% CI 0.27–1.50; *P* = 0.307; *I*^2^ = 72.7%; *P*_H_ = 0.026; Figure 5).

### Subgroup Analysis

The results of subgroup analyses are shown in Table 2. Compression therapy was significantly associated with a reduction in the incidence of PTS in both the subgroups when stratified by the occurrence of DVT and interval instead of other stratification factors.

**TABLE 2:**
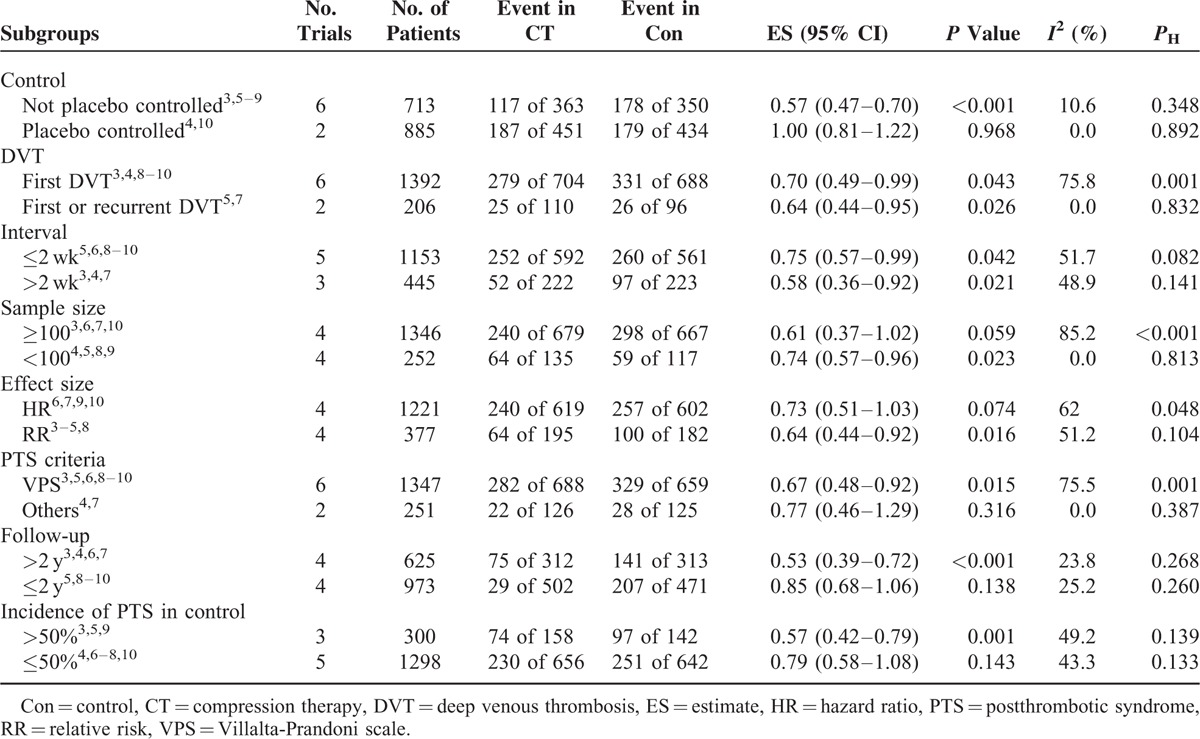
Subgroup Analysis According to Various Categories for PTS

### Sensitivity Analysis

Table 3 summaries the results of sensitivity analysis according to various inclusion criteria. Any single study could not substantially alter the pooled estimate, with a narrow range from 0.60 (95% CI 0.49–0.75) to 0.77 (95% CI 0.62–0.96).

**TABLE 3:**
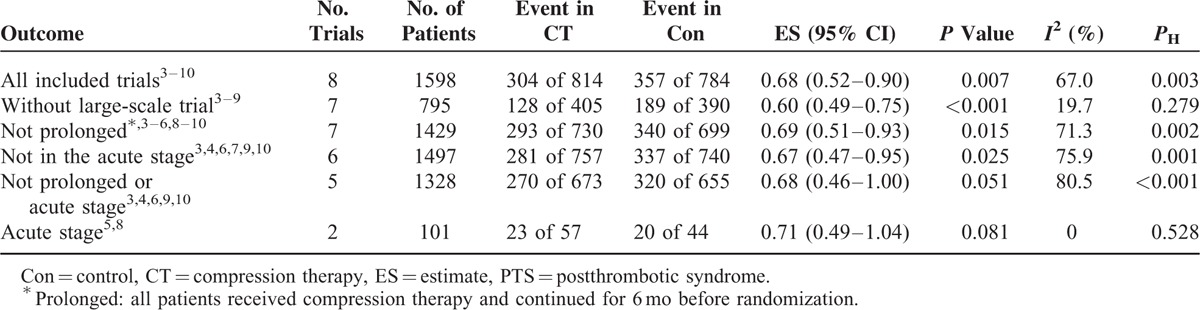
Sensitivity Analysis According to Various Inclusion Criteria for PTS

### The Secondary Outcomes

Compression therapy had no impacts on the incidence of recurrent venous thromboembolism (RR 0.91, 95% CI 0.65–1.27; *P* = 0.575; *I*^2^ = 0.0%; *P*_H_ = 0.811; Figure S2, Additional file 5, http://links.lww.com/MD/A356), the incidence of ulceration (RR 0.74, 95% CI 0.36–1.53; *P* = 0.422; *I*^2^ = 12.3%; *P*_H_ = 0.320; Figure S3, Additional file 6, http://links.lww.com/MD/A356), or the mortality (RR 0.99, 95% CI 0.72–1.37; *P* = 0.956; *I*^2^ = 0.0%; *P*_H_ = 0.408; Figure S4, Additional file 7, http://links.lww.com/MD/A356).

### Publication Bias

No publication bias was detected by visually inspecting funnel plot and Egger test with a *P* value of 0.836, indicating a low likelihood of publication bias. However, the power of test was low because of limited studies included.

## DISCUSSION

### Main Findings

Our further systematic review and meta-analysis suggested that compression therapy could significantly reduce the incidence of PTS, but only the incidence of mild/moderate PTS instead of severe PTS. Furthermore, compression therapy had no effects on the incidence of recurrent venous thromboembolism, the incidence of ulceration, or the mortality.

### Comparison With the Previous Studies

Our findings are partly consistent with the previous meta-analyses^18,19,31^ and further extend them in several important ways. This meta-analysis reinforced earlier results by adding 3 recently published RCTs with 936 cases,^8–10^ containing more than twice the PTS events of the previous meta-analysis.^19^ Additionally, subgroup analysis and sensitivity analysis validated the robustness of the pooled estimate. Moreover, other important clinical outcomes like the incidence of recurrent venous thromboembolism, the incidence of ulceration, and the mortality were also researched to give a comprehensive evaluation of compression therapy.

A large-scale trial enrolling 806 participants included in the meta-analysis suggested compression therapy failed to prevent PTS (HR 1.00, 95% CI 0.81–1.24). It is noteworthy that the substantial heterogeneity was caused by this trial according to forest plots and sensitivity analysis. The contradictory conclusion and heterogeneity might be attributed to following aspects. First, placebo-controlled design was adopted in this trial and the other one,^4^ and both of them gave a null result. Placebo-controlled design could certainly protect against bias inherent to open trials, especially when the outcome is subjective. However, it remains unclear whether the placebo contributes to the prevention of PTS or not, and it may counteract the preventive effect. Second, considering the important role of anticoagulants and great improvements made in recent years, the effect of compression therapy might have been hypothesized to be masked by anticoagulant treatment. Third, the compliance was very low with only 55.6% of participants wearing compression stockings for ≥3 d/wk, whereas the compliances of previous trials were about 90%.^3,6^ The low compliance resulting from unknown reasons might also contribute to the inefficacy. Though limitations existed, the contradictory conclusion from this trial challenged the traditional concept of compression therapy in the prevention of PTS, leaving some living issues: is there any benefit of prevention with compression therapy? Is the compression therapy necessary with the improvement of anticoagulants? Given the substantial heterogeneity and inconsistence with the large-scale trial, the conclusion of our meta-analysis should be interpreted with caution.

Another 2 studies assessing the effect of compression therapy on patients with DVT were included in our systematic review.^29,30^ Both concluded that compression therapy could lead to an immediately pronounced reduction of pain. Moreover, the clinical score and relief of swelling were observed to be significantly better in the compression group. The conference abstract^30^ found that compression therapy could enhance the thrombus reduction, which was unfavorable for the development of PTS. However, no subjective benefit was observed with enhanced thrombus reduction. Therefore, we hypothesize that the controversial effects of compression therapy on PTS might be due to the easily affected characteristic of subjective outcome.

Analysis of PTS severity category suggested that compression therapy could reduce the incidence of mild/moderate PTS but not the incidence of severe PTS. The null results for severe PTS were not conclusive inasmuch as only 4 trials were included^3,5,6,10^ and this pooled effect was substantially altered when removing the large-scale one (RR 0.44, 95% CI 0.25–0.79).^10^ It might be because VPS was oversensitive to mild/moderate PTS and less sensitive to severe PTS.^32,33^ Additionally, the low incidence of severe PTS also contributed to the nonsignificant result, as confirmed by our subgroup analysis according to the incidence of PTS. Considering the limitations of PTS diagnostic criteria and great discrepancy with the large-scale trial, the ineffectiveness of compression therapy on severe PTS should also be treated cautiously.

### Subgroup Analysis and Sensitivity Analysis

In subgroup analysis, the results suggested that the pooled effect could be affected by control, sample size, ES, PTS criteria, follow-up, the incidence of PTS in control rather than the occurrence of DVT and interval between diagnosis and treatment. Placebo could also bring benefits,^34^ especially in measurement of subjective symptoms. As PTS is defined partially based on subjective symptoms, the null association in comparison with placebo subgroups could be due to, at least partly, the placebo effect. The discrepancy of subgroups with respect to the sample size might be explained by the determinant role of the large-scale trial, as substantial heterogeneity and critical ES (ES 0.61, 95% CI 0.37–1.02) in the subgroup of sample size >100 were observed. The inconsistent subgroup results regarding PTS definition might be caused by the poor agreement and different sensitivity to various categories of PTS between VPS and Ginsberg measure.^32^ The negative association in the subgroup of HRs could be explained by the substantial heterogeneity and null association from the large-scale trial. Though the beneficial effect was not observed in the subgroup of intervention in the acute stage, several studies, with relative small sample sizes (range from 31 to 64), reported that compression therapy could reduce leg swelling and pain much faster and more effectively in the acute stage.^5,8,29,30^ Therefore, its preventive efficacy could not be completely ruled out considering the critical nonsignificant level (ES 0.71, 95% CI 0.49–1.04) and small sample size of 101. The cumulative incidence of PTS increases even 20 years after DVT, thus longer follow-up is usually accompanied with increased incidence of PTS.^1,35^ Additionally, the results of subgroup analysis of long follow-up and high incidence of PTS consistently revealed the efficacy of compression therapy. However, results of subgroup analysis of short follow-up and low incidence did not confirm this promising finding, indicating that unobserved benefit of compression therapy may be due to inadequate duration of follow-up. Suffering from limitations of their observational investigation and the decreased statistical power, these subgroup and sensitivity analyses should be interpreted with cautions.

### Limitations

Several limitations of our study merit consideration. First, considerable heterogeneity was detected among studies. To assess the impact of various clinical factors on the pooled estimate and explore the source of heterogeneity, subgroup and sensitivity analyses had been conducted, and potential source had been found. Second, in the subgroup and sensitivity analyses, the results were inconsistent. However, the interpretation of these analyses should be interpreted with cautions due to their limitations. Third, the outcome based on subjective symptoms was more susceptible to errors arising from bias in an open-labeled design. Our findings largely relied on this type of studies, and the ascertainment of PTS was defined mainly on subjective symptoms. Fourth, both HR and RR were used in our meta-analysis. RR was subjected to selection bias regarding endpoints by comparison of HR. Though HR was preferred to be used in our meta-analysis, HR could only be extracted in 3 studies^6,7,10^ and estimated from the survival curves in 1 study.^9^ Finally, given limited amount of studies included and unavailability of confounding factors, we could not give a comprehensive and detailed evaluation of the secondary outcomes. Thus, these outcomes should be treated cautiously.

In summary, the present systematic review and meta-analysis suggests that compression therapy could effectively prevent PTS, and current evidence still supports compression therapy to be a clinical practice for prophylaxis of PTS in adult patients after DVT. However, our findings should be interpreted with caution due to heterogeneity and hence more large-scale and well-designed RCTs are still warranted.
